# Structural connectivity and brain network analyses in Parkinson's disease: A cross-sectional and longitudinal study

**DOI:** 10.3389/fneur.2023.1137780

**Published:** 2023-03-23

**Authors:** Maurizio Bergamino, Elizabeth G. Keeling, Nicola J. Ray, Antonella Macerollo, Monty Silverdale, Ashley M. Stokes

**Affiliations:** ^1^Barrow Neuroimaging Innovation Center, Barrow Neurological Institute, Phoenix, AZ, United States; ^2^School of Life Sciences, Arizona State University, Tempe, AZ, United States; ^3^Health, Psychology and Communities Research Centre, Department of Psychology, Manchester Metropolitan University, Manchester, United Kingdom; ^4^Neurology Department, The Walton Centre NHS Foundation Trust, Liverpool, United Kingdom; ^5^Institute of Systems, Molecular and Integrative Biology, School of Life Sciences, University of Liverpool, Liverpool, United Kingdom; ^6^Manchester Centre for Clinical Neurosciences, University of Manchester, Manchester, United Kingdom

**Keywords:** Parkinson's disease, Parkinson's Progression Markers Initiative, structural connectivity, diffusion magnetic resonance imaging, network analysis

## Abstract

**Introduction:**

Parkinson's disease (PD) is an idiopathic disease of the central nervous system characterized by both motor and non-motor symptoms. It is the second most common neurodegenerative disease. Magnetic resonance imaging (MRI) can reveal underlying brain changes associated with PD.

**Objective:**

In this study, structural connectivity and white matter networks were analyzed by diffusion MRI and graph theory in a cohort of patients with PD and a cohort of healthy controls (HC) obtained from the Parkinson's Progression Markers Initiative (PPMI) database in a cross-sectional analysis. Furthermore, we investigated longitudinal changes in the PD cohort over 36 months.

**Result:**

Compared with the control group, participants with PD showed lower structural connectivity in several brain areas, including the corpus callosum, fornix, and uncinate fasciculus, which were also confirmed by a large effect-size. Additionally, altered connectivity between baseline and after 36 months was found in different network paths inside the white matter with a medium effect-size. Network analysis showed trends toward lower network density in PD compared with HC at baseline and after 36 months, though not significant after correction. Significant differences were observed in nodal degree and strength in several nodes.

**Conclusion:**

In conclusion, altered structural and network metrics in several brain regions, such as corpus callosum, fornix, and cingulum were found in PD, compared to HC. We also report altered connectivity in the PD group after 36 months, reflecting the impact of both PD pathology and aging processes. These results indicate that structural and network metrics might yield insight into network reorganization that occurs in PD.

## Introduction

Parkinson's disease (PD) is an idiopathic disease of the central nervous system characterized by both motor and non-motor symptoms. It is the second most common neurodegenerative disease worldwide with rising incidence and prevalence, partly due to changing population demographics (1), with world-wide PD cases expected to increase from an estimated 4.1 million in 2005 to ~8.7 million by 2030 (2). Biomarkers play a critical role in the management of patients with PD, including for diagnosis and monitoring disease progression. Neuroimaging techniques may provide such biomarkers, but these must be investigated in large-scale, multicenter, and longitudinal samples. For example, the Parkinson's Progression Markers Initiative (PPMI) study (3) (https://www.ppmi-info.org/) is a comprehensive observational, international, multicenter study designed to identify PD progression biomarkers, both to improve understanding of disease etiology and course and to provide crucial tools to evaluate the efficacy of PD-modifying therapeutics.

In recent years, PD has been studied with a range of MRI techniques, such as structural imaging, functional MRI (fMRI), and diffusion weighted MRI (dMRI). These have been shown to be useful not only in PD but also in other forms of parkinsonism (4). For instance, dMRI has been used extensively to study structural changes in brain white matter (WM), such as axonal caliber, density, myelination, and orientation, along with WM-dependent structural connectivity in early PD (5). One of the most used dMRI analysis methods that can help to understand the pathophysiology and evolution of PD is diffusion tensor imaging (DTI) (6–9). A recent meta-analysis of DTI findings showed consistent differences indicative of neurodegeneration between PD and healthy controls (HC) in subcortical regions (including the substantia nigra), cingulate and temporal cortex, and the corpus callosum (10). Paradoxical changes in the corticospinal tract were also observed and could be indicative of either compensatory changes or selective neurodegeneration of proximate WM fibers. The latter highlights a known limitation related to standard DTI, notably that DTI is not able to resolve crossing fibers within brain voxels (11). To overcome this limitation, more advanced dMRI methods must be used (12). For example, the distribution of fiber orientations (i.e., fiber orientation distribution, FOD) within a voxel can be directly estimated from dMRI data without prior assumptions regarding the number of fiber populations present (13).

Combining dMRI and tractography enables the assessment of brain structural connectivity, which represents the WM tracts that physically interconnect brain regions *in vivo* (14). Additionally, tractography, in combination with graph theory, can been used to reconstruct and analyze the structural whole-brain connectivity in PD (10, 15), as well as other neurodegenerative disorders (16, 17). These studies in PD have revealed the existence of novel PD-specific anatomic networks, characterized by alterations in cortical–subcortical loops, reduced modular organization, and compensatory changes (15, 18).

In the current study, structural connectivity and WM networks were analyzed using the FOD from dMRI and graph theory in a cohort of patients with PD and a cohort of HCs recruited to the PPMI study (3). To examine whether structural connectivity and WM network analysis could yield information about PD and its progression, we investigated the differences in structural connectivity and WM networks between these groups in a cross-sectional analysis. We also investigated longitudinal changes in the PD cohort over 36 months.

## Method

### Subjects and cognitive/neuropsychiatric testing

A total of 161 subjects from the PPMI database were included in this study. The subjects were divided in two groups: 75 HCs (26 females; age 59.7 ± 10.5 years) and 86 PD (29 females; age 61.1 ± 9.8 years). We included PD participants with a minimum of 12 months of disease duration (mean disease duration: 19.0 ± 7.4 years) available in the PPMI database. In this study, follow-up was analyzed after 36 months. The inclusion criteria for recruitment into the PPMI study as a participant with PD or HC can be found here: https://www.ppmi-info.org/study-design/study-cohorts. In addition, to reduce variability in images due to scanner differences, we selected only participants who had images collected on a Siemens 3T TrioTim.

A comprehensive baseline clinical evaluation of cognitive, behavioral, and motor assessment was performed for every participant by the site investigators at the enrolment time. Motor severity score and global assessment of cognition were calculated for each participant using the Movement Disorder Society sponsored-Unified Parkinson's Disease Related Scale Part III scores (MDS-UPDRS)-III (19) and the Montreal Cognitive Assessment (MoCA) (20), respectively. The functional disability associated with PD was evaluated by the Hoehn and Yahr (H&Y) scale (21). In addition, depressive symptoms were identified using the Geriatric Depression (GD) Rating Scale (22). Clinical and demographic characteristics are reported in Table 1.

**Table 1:** Complete subject characteristics.

	***N* tot (F)**	**<Age> (S.D.) [y]**	**Motion (SD) [mm]**	**Removed (F) [motion > 3 mm]**				
**HC**	75 (26)	59.7 (10.5)	1.35 (1.11)	6 (1)				
**PD**	86 (29)	61.1 (9.8)	1.94 (2.16)	11 (4)				
*t*-test		*t* = 0.785; *p* = 0.383	*t* = 2.132; *p* = 0.035					
**Analysis**								
			**Baseline**					
	***N*** **(F)**	<**Age**> **(S.D.) [year]**	**Motion (SD) [mm]**	**Disease duration (months)**	<**MDS-UPDRS**> **[*****n*****]**	<**H&Y**> **[*****n*****]**	<**MoCA**> **[*****n*****]**	<**GD score**> **(S.D.) [*****n*****]**
HC	69 (25)	60.0 (10.6)	1.29 (1.58)	**–**	1.51 (1.87) [47]	–	28.05 (1.11) [58]	1.40 (2.71) [69]
PD	75 (25)	61.5 (9.8)	1.76 (2.08)	19.03 (7.43)	20.17 (9.32) [75]	1.53 (0.50) [75]	27.45 (2.17) [75]	2.24 (2.47) [54]
*t*-test		*t* = 0.882; *p* = 0.379	*t* = 1.517; *p* = 0.132					
Mann Whitney test					*W* = 14.5; *p* < 0.001		*W* = 2,380; *p =* 0.274	*W* = 1,617; *p* < 0.001

F, females; SD, standard deviation; MDS-UPDRS, movement disorder society sponsored-unified Parkinson's disease related scale part III scores; H&Y, Hoehn and Yahr Scale; MoCA, montreal cognitive assessment; GD, Geriatric Depression Rating Scale. [n] was the number of the subjects where scores were available.

### MRI data processing

MRI data were downloaded from the PPMI database (3). The dMRI acquisition was performed using 64 diffusion-encoding directions (*b*-value = 1,000 s/mm^2^) and one non-diffusion weighted acquisition (b0 image), with acquisition parameters: TR/TE = 800/88 ms, matrix = 116 × 116, voxel sizes = (2.0 × 2.0 × 2.0) mm, and flip-angle = 90°. High resolution T1-weighted (T1-w) images were acquired using a 3D sagittal magnetization prepared rapid gradient echo (MP-RAGE) sequence with TR/TE = 1,970/3.17 ms, matrix = 256 × 256, voxel sizes = (1.0 × 1.0 × 1.0) mm, and flip-angle = 15°.

All DICOM dMRI images were converted to NIFTI format using dcm2niix (https://github.com/rordenlab/dcm2niix) and were preprocessed using MRtrix3 (23), FSL (24), AFNI (https://afni.nimh.nih.gov), and the Advanced Normalization Tool (ANTs; http://stnava.github.io/ANTs/). Pre-processing steps included (a) denoising by *dwidenoise* (MRtrix3), (b) alignment and eddy-currents corrections by *eddy* (FSL), and (c) bias field correction (ANTs). The eddy quality control (QC) tools (25) were used to evaluate the quality of each dMRI dataset. Slices with signal loss caused by subject movement coinciding with the diffusion encoding were detected and replaced by predictions made by a Gaussian process. To increase anatomical contrast and improve downstream template generation, registration, tractography, and statistics, the pre-processed dMRI images were upsampled to 1.25 mm by *mrgrid* (MRtrix3). Subsequently, brain extraction on the upsampled B0 images was computed by *dwi2mask* (MRtrix3). Using the MP-RAGE images [intensity normalized by *3dUnifize* (AFNI) and brain extracted using Robust Brain Extraction (ROBEX) (26)], a five-tissue-type (5TT) segmented tissue image—suitable for use in Anatomically-Constrained Tractography (ACT)—was generated by the “FSL” algorithm (27) and coregistered to the upsampled dMRI space using non-linear coregistration (ANTs). We then estimated the response function(s) for spherical deconvolution using *dwi2response* (MRtrix3), while the FOD was estimated by *dwi2fod* (MRtrix3) (13). Tractography was performed by *tckgen* (MRtrix3) with a second-order integration over FOD (iFOD2) algorithm (28) by using the 5TT images previously generated, seeded from the gray matter/white matter interface, and 5 million streamlines (min. length 4 mm, max. length = 200 mm, unidirectional tracking, maximum angle in degrees between successive steps = 45 (default) with backtrack option). The connectomes [*tck2connectome;* (MRtrix3)] were generated from the automated anatomical labeling (AAL) atlas (29) and were subsequently filtered by removing “non-connecting” streamlines. Additionally, the *tcksift2* algorithm (MRtrix3) (30) was used to optimize per-streamline cross-section multipliers to match a whole-brain tractogram to fixel-wise fiber densities.

### Network analysis

Global efficiency (which corresponds to the average of the inverse of the shortest path length in the network), assortativity coefficient (correlation coefficient between the degrees of all nodes on two opposite ends of a link), network density (the fraction of present connections to possible connections), and the mean strength were evaluated. Additionally, for each node, we assessed the betweenness centrality (the number of shortest paths that pass through a node, with high betweenness centrality values indicating that more passages traverse a node), the nodal degree (a measure of how connected each node is), and the strength. These metrics were directly retrieved from structural connectivity matrices using the Brain Connectivity Toolbox (BCT) in MATLAB (MathWorks, Natick, MA, United States) (31). For this analysis, weighted networks were used.

### Statistical analysis

In Table 1, age, motion during dMRI scan, disease duration, MDS-UPDRS, H&Y, MoCA, and GD score are presented as mean and standard deviation (SD) for each group. Differences in age and motion were evaluated by the Student's *t*-test. Differences in cognitive test scores were assessed using Mann–Whitney tests.

Structural connectivity and network analysis metrics were compared between PD and HC cross-sectionally *via* analysis of covariance (ANCOVA), with age and gender as covariates. Paired *t*-tests, corrected for age, were used to compare structural and network metrics in participants with PD at baseline and 36-months.

For structural connectivity metrics, significance was determined at a *p* < 0.05, with a family-wise error (FWE) correction for multiple comparisons. FWE correction was also used to correct the *p*-values from the networks analysis.

For all analyses, an in-house R script (http://www.R-project.org) was used to calculate the Cohen'd effect size for both cross-sectional and longitudinal analyses (32) (large effect at *d* > 0.80 and medium effect at *d* > 0.50). BrainNetViewer (33) (https://www.nitrc.org/projects/bnv) was used to visualize structural connectivity and network analyses. The JHU DTI-based white-matter atlases (34) was used to identify regions where WM connectivity and networks changed longitudinally.

Correlations between structural connectivity and MDS-UPDRS/MoCA scores were analyzed by a linear model with age and sex as covariates. FWE correction was used to correct all *p*-values.

## Results

### Baseline clinical and demographic outcomes

Significant differences in motion during the dMRI scan were found between HC and PD (*t* = 2.132; *p* = 0.035). As subject motion during the dMRI acquisition can adversely impact the results, subjects with an average motion >3 mm were excluded from further analysis. Specifically, six HC (one female) and 11 PD (four females) were removed from the final statistical analysis. The final statistical analysis included 69 HC (25 females, baseline only) and 75 PD (25 females). The groups did not differ significantly in motion during dMRI acquisition (*t* = 1.517; *p* = 0.132), in age (*t* = 0.882; *p* = 0.379), or in MoCA scores (*W* = 2,380; *p* = 0.274). The groups did differ in MDS-UPDRS (*W* = 14.5; *p* < 0.001) and in GDS score (*W* = 1,617; *p* < 0.001). Complete clinical and demographic comparisons are shown in Table 1.

### Baseline comparisons (HC vs. PD) between structural and network metrics

**Figure 1:**
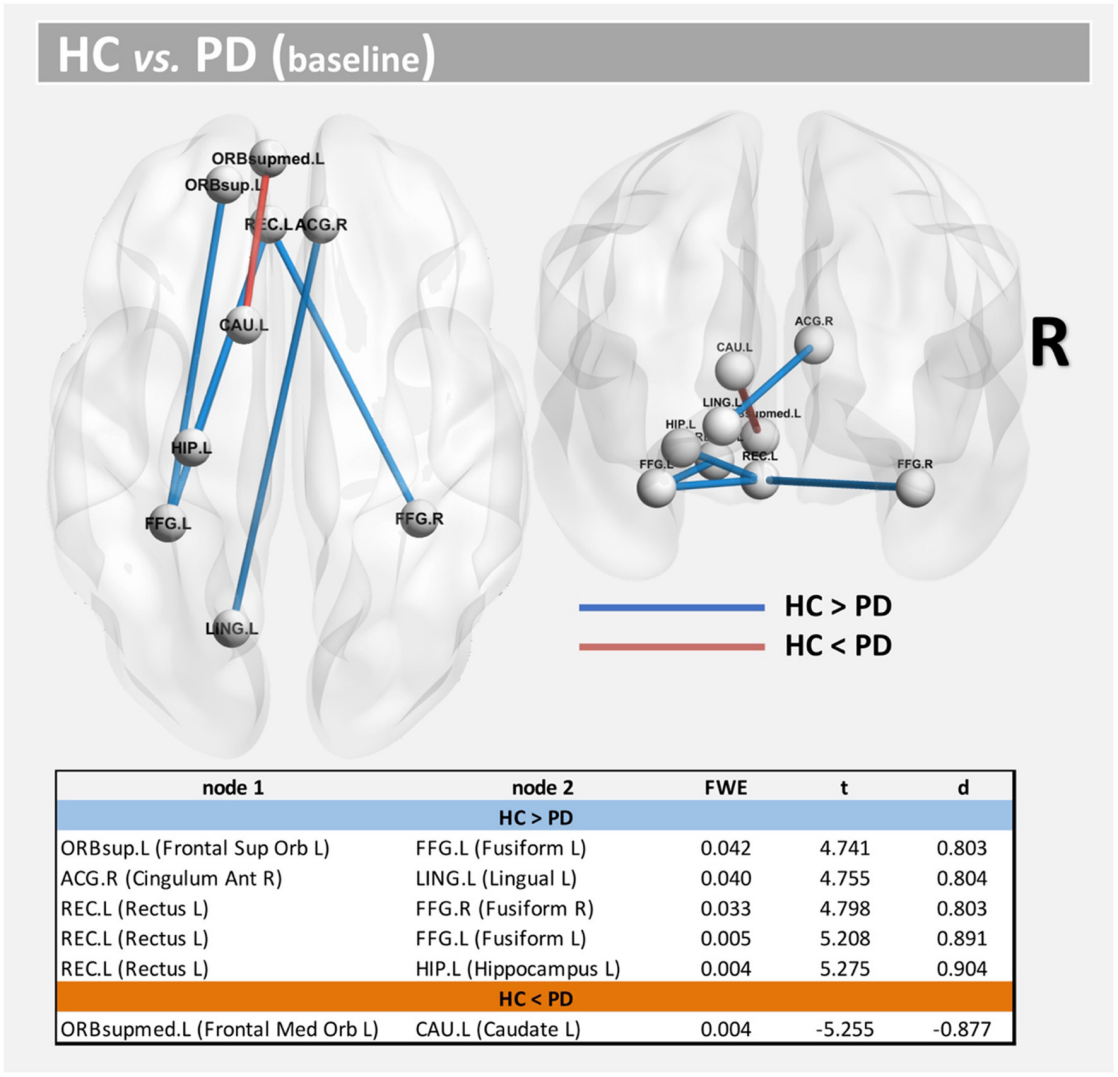
Altered structural connectivity between HC and PD at baseline (cross-sectional analysis). Compared with HCs, PD showed lower connectivity across different nodes (Paths in blue color). However, higher connectivity in PD was also found between nodes CAU.L and ORBsupmed.L (Paths in red color). See also Table 2. For all significant paths we found FWE <0.05 and large effect-size.

Using both FWE <0.05 and large effect-size, we found higher structural connectivity in HC than PD across several nodes (Figure 1, paths in blue color): ORBsup.L [left superior frontal gyrus (orbital part)] and FFG.L (left fusiform) (*t* = 4.741; *d* = 0.803), ACG.R (right anterior cingulum) and LING.L (left lingual) (*t* = 4.755; *d* = 0.804), REC.L (left rectus) and FFG.R (right fusiform) (*t* = 4.798; *d* = 0.803), REC.L (left rectus) and FFG.L (left fusiform) (*t* = 5.208; *d* = 0.891), and HIP.L (left hippocampus) and REC.L (left rectus) (*t* = 5.275; *d* = 0.904). Regions with altered structural connectivity included the corpus callosum, fornix, sagittal stratum, external capsule, cingulum, and uncinate fasciculus (see Table 2).

**Table 2:** WM areas where we found altered structural connectivity between HC and PD at baseline (cross-sectional analysis), as indicated by ✓.

**HC** > **PD (baseline)**					
**WM area**	**ORBsup.L—FFG.L**	**ACG.R—LING.L**	**REC.L—FFG.R**	**REC.L—FFG.L**	**REC.L—HIP.L**
Genu of corpus callosum	**–**	✓	✓	✓	✓
Body of corpus callosum	**–**	✓	**–**	**–**	**–**
Splenium of corpus callosum	**–**	✓	**–**	**–**	**–**
Fornix (column and body of fornix)	**–**	✓	✓	✓	✓
Anterior limb of internal capsule L	**–**	✓	**–**	**–**	✓
Anterior corona radiata L	✓	**–**	**–**	✓	✓
Posterior thalamic radiation L	✓	✓	**–**	✓	**–**
Sagittal stratum R	**–**	**–**	✓	**–**	**–**
Sagittal stratum L	✓	✓	**–**	✓	✓
External capsule R	**–**	**–**	✓	**–**	**–**
External capsule L	✓	✓	**–**	✓	✓
Cingulum (cingulate gyrus) R	**–**	✓	**–**	**–**	**–**
Cingulum (cingulate gyrus) L	**–**	✓	**–**	**–**	**–**
Cingulum (hippocampus) L	**–**	✓	**–**	**–**	**–**
Fornix (cres)/stria terminalis R	**–**	**–**	✓	**–**	**–**
Fornix (cres)/stria terminalis L	✓	✓	**–**	✓	✓
Uncinate fasciculus R	**–**	**–**	✓	**–**	**–**
Uncinate fasciculus L	✓	✓	**–**	✓	✓
**HC**<**PD (baseline)**					
**WM Area**	**ORBsupmed.L—CAU.L**				
Posterior limb of internal capsule R	✓				
Anterior corona radiata L	✓				

We found for all significant paths an FWE <0.05 and large effect-size. The dash (–) indicates regions that did not have significant differences.

**Figure 2:**
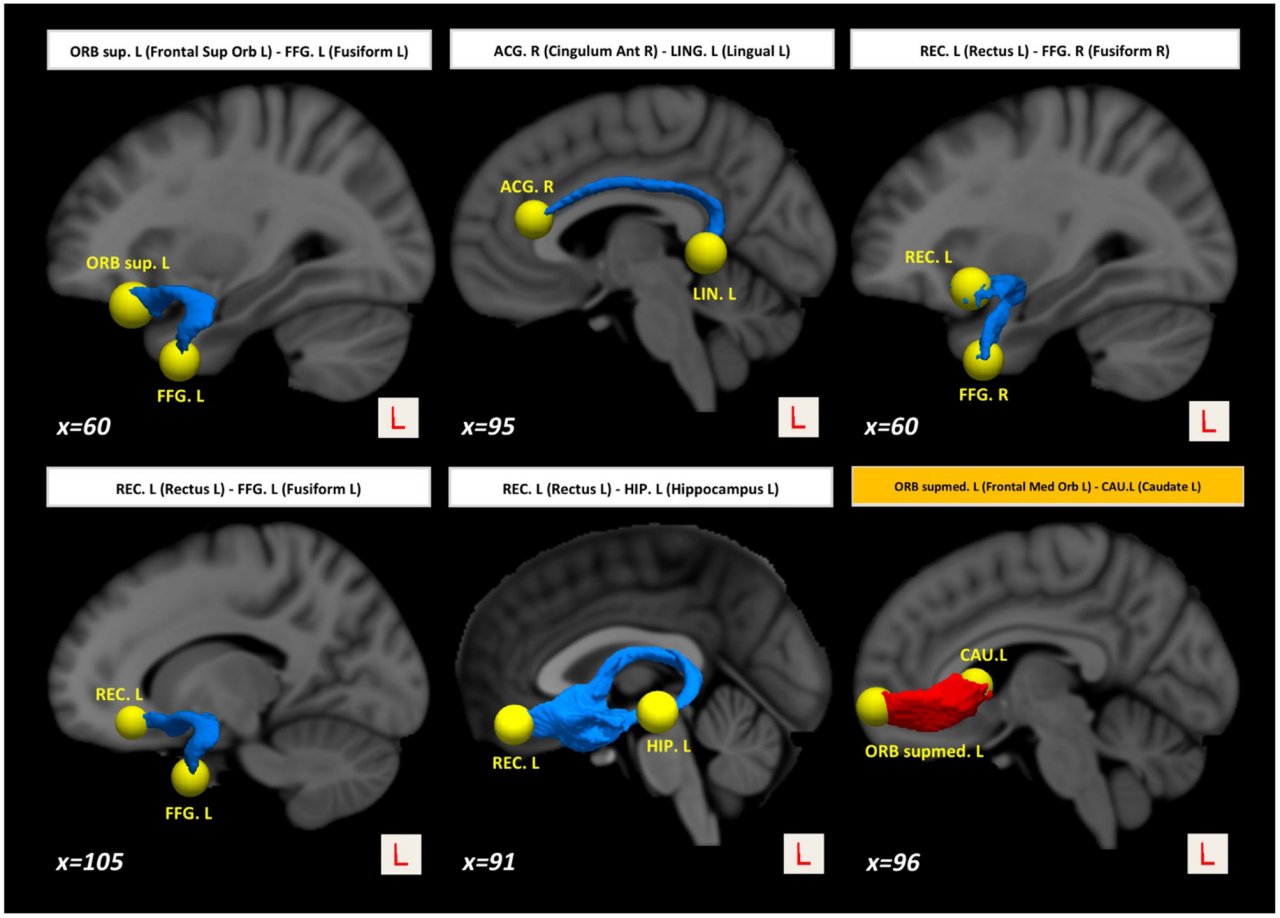
Tract reconstructions corresponding to structural connectivity differences between HC and PD at baseline. The tracts were reconstructed by *connectome2tck* (MRtrix3) in the native space for all HC subjects, converted to track-weighted image by *tckmap* (MRtrix3), and normalized and averaged in the MNI 1mm standard space. For the visualization, Trackvis software (https://www.trackvis.org) was used. The blue tracts show higher connectivity in HC than PD. The red tracts show higher connectivity in PD than HC.

Interestingly, we also found higher structural connectivity in PD compared with the control group (Figure 1, paths in red color) between nodes CAU.L (left caudate) and ORBsupmed.L [left medial frontal gyrus (orbital part)] (*t* = 5.255; *d* = 0.877). WM regions associated with this altered connectivity included the right posterior limb of internal capsule and the left anterior corona radiata (see Table 2). Figure 2 shows the tract reconstructions for this analysis.

**Figure 3:**
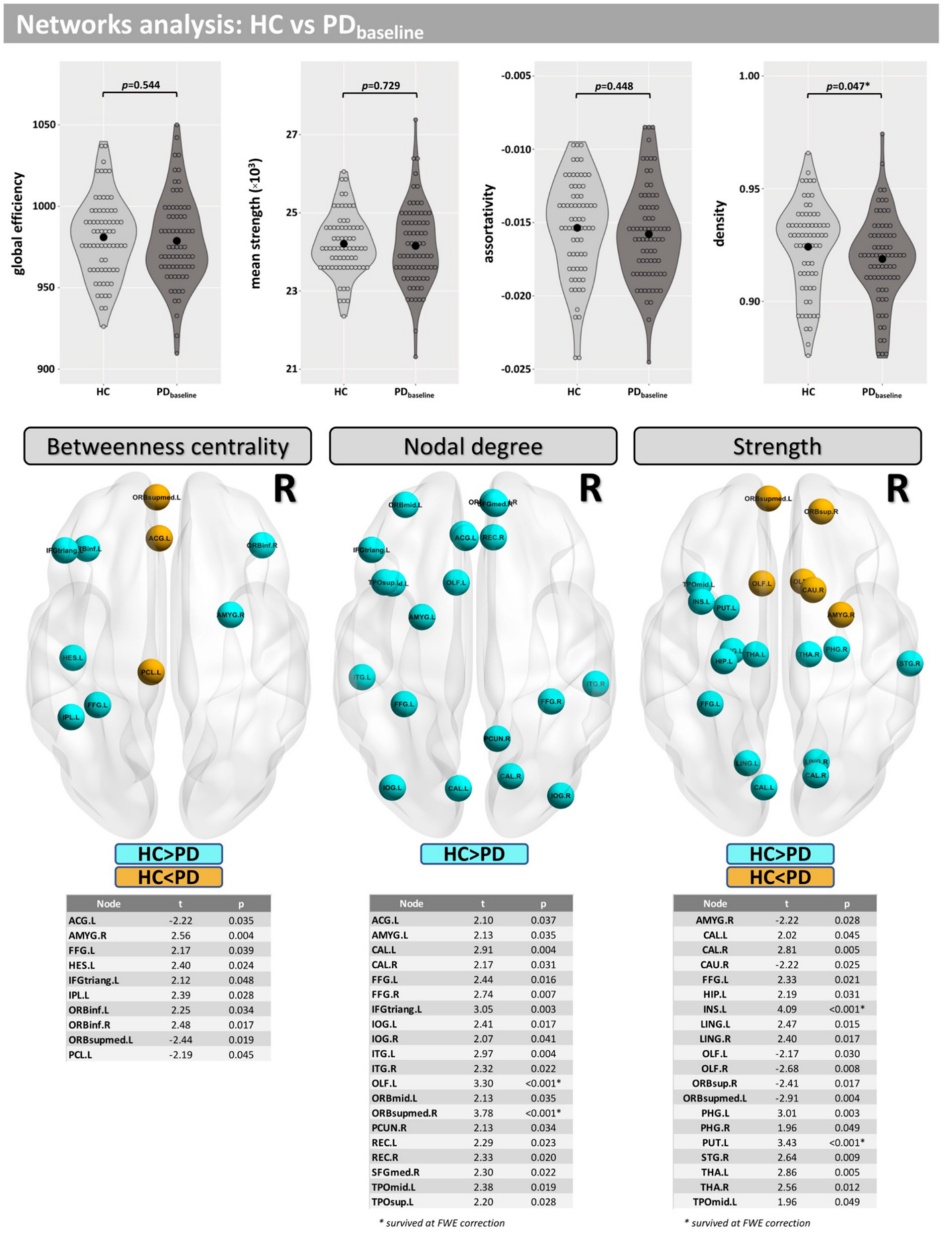
Network analysis for the cross-sectional part of this study. Differences between groups were found for the network density and in several nodes for “betweenness centrality,” “nodal degree,” and “strength.”

Network density was found to be lower in the PD group, but this finding did not survive correction for multiple comparisons (*p* = 0.047-uncorrected). Additionally, differences were found at FWE <0.05 in betweenness centrality (mainly located inside the insula, frontal, and temporal lobes), nodal degree (mainly located inside the caudate, occipital, and temporal lobes) and strength (mainly located inside the caudate, insula, putamen, thalamus, occipital, and temporal lobes) (Figure 3).

### Changes in structural and network metrics over 36 months in participants with PD

**Figure 4:**
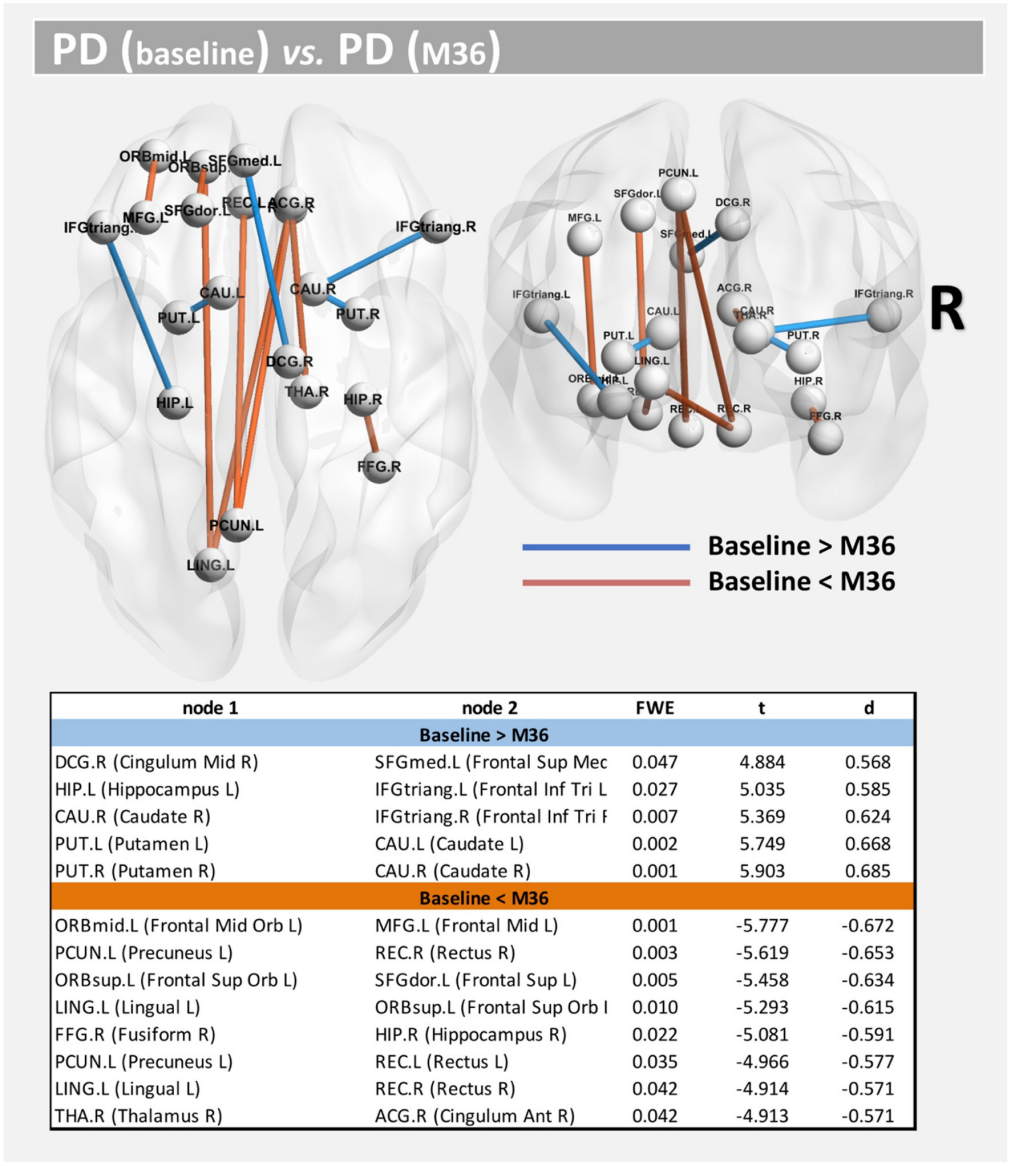
Altered structural connectivity in PD between baseline and M36 (longitudinal analysis). Compared with baseline, PD subjects at M36 showed higher connectivity (red paths) and lower connectivity (blue paths) across different nodes; see also Table 3. For all significant paths we found FWE <0.05 and medium effect-size.

Compared with baseline, several paths with higher connectivity were found in PD after 36 months (Figure 4, paths in red color). However, in this case, all significant paths showed a medium effect-size (between 0.571 and 0.672). The main WM areas associated with this trend and that show an increase in connectivity after 36 months included the genu and body of the corpus callosum, fornix, left posterior thalamic radiation, sagittal stratum, left external capsule, and left uncinate fasciculus (see Table 3).

**Table 3:** WM areas where we found altered sstructural connectivity in PD between baseline and M36 (longitudinal analysis), as indicated by ✓.

**PD (M36)** > **PD (baseline)**								
**WM area**	**ORBmid.L—MFG.L**	**PCUN.L—REC.R**	**ORBsup.L—SFGdor.L**	**LING.L–ORBsup.L**	**FFG.R–HIP.R**	**PCUN.L–REC.L**	**LING.L–REC.R**	**THA.R–ACG.R**
Genu of corpus callosum	**–**	✓	**–**	**–**	**–**	✓	**–**	✓
Body of corpus callosum	**–**	✓	**–**	**–**	**–**	✓	**–**	**–**
Splenium of corpus callosum	**–**	**–**	**–**	**–**	**–**	✓	**–**	**–**
Fornix (column and body of fornix)	**–**	✓	**–**	**–**	**–**	✓	✓	✓
Anterior limb of internal capsule R	**–**	**–**	**–**	**–**	**–**	**–**	**–**	✓
Retrolenticular part of internal capsule L	**–**	✓	**–**	✓	**–**	✓	**–**	**–**
Anterior corona radiata R	**–**	**–**	**–**	**–**	**–**	**–**	**–**	✓
Anterior corona radiata L	**–**	**–**	**–**	✓	**–**	✓	**–**	**–**
Posterior corona radiata L	**–**	**–**	**–**	**–**	**–**	✓	**–**	**–**
Posterior thalamic radiation L	**–**	**–**	**–**	✓	**–**	✓	✓	**–**
Sagittal stratum R	**–**	**–**	**–**	**–**	✓	**–**	**–**	**–**
Sagittal stratum L	**–**	**–**	**–**	✓	**–**	✓	✓	**–**
External capsule L	**–**	**–**	**–**	✓	**–**	✓	✓	**–**
Cingulum (cingulate gyrus) L	**–**	✓	**–**	**–**	**–**	✓	**–**	**–**
Cingulum (hippocampus) R	**–**	**–**	**–**	**–**	✓	**–**	**–**	**–**
Fornix (cres)/stria terminalis R	**–**	**–**	**–**	**–**	✓	**–**	**–**	**–**
Fornix (cres)/stria terminalis L	**–**	**–**	**–**	**–**	**–**	✓	✓	**–**
Superior fronto-occipital fasciculus R	**–**	**–**	**–**	**–**	**–**	**–**	**–**	✓
Uncinate fasciculus L	✓	**–**	✓	✓	**–**	✓	✓	**–**
**PD (M36)**<**PD (baseline)**								
**WM Area**	**DCG.R—SFGmed.L**	**HIP.L—IFGtriang.L**	**CAU.R—IFGtriang.R**	**PUT.L—CAU.L**	**PUT.R—CAU.R**			
Genu of corpus callosum	✓	**–**	**–**	**–**	**–**			
Body of corpus callosum	✓	**–**	**–**	**–**	**–**			
Anterior limb of internal capsule R	**–**	**–**	✓	**–**	✓			
Anterior limb of internal capsule L	**–**	✓	**–**	✓	**–**			
Anterior corona radiata R	✓	**–**	✓	**–**	✓			
Anterior corona radiata L	✓	✓	**–**	**–**	**–**			
Sagittal stratum L	**–**	✓	**–**	**–**	**–**			
External capsule R	**–**	**–**	**–**	**–**	✓			
External capsule L	**–**	✓	**–**	✓	**–**			
Cingulum (cingulate gyrus) R	✓	**–**	**–**	**–**	**–**			
Cingulum (cingulate gyrus) L	✓	**–**	**–**	**–**	**–**			
Fornix (cres)/stria terminalis L	**–**	✓	**–**	**–**	**–**			
Superior fronto-occipital fasciculus R	**–**	**–**	✓	**–**	✓			
Superior fronto-occipital fasciculus L	**–**	✓	**–**	✓	**–**			

We found for all significant paths an FWE <0.05 and medium effect-size. The dash (–) indicates regions that did not have significant differences.

**Figure 5:**
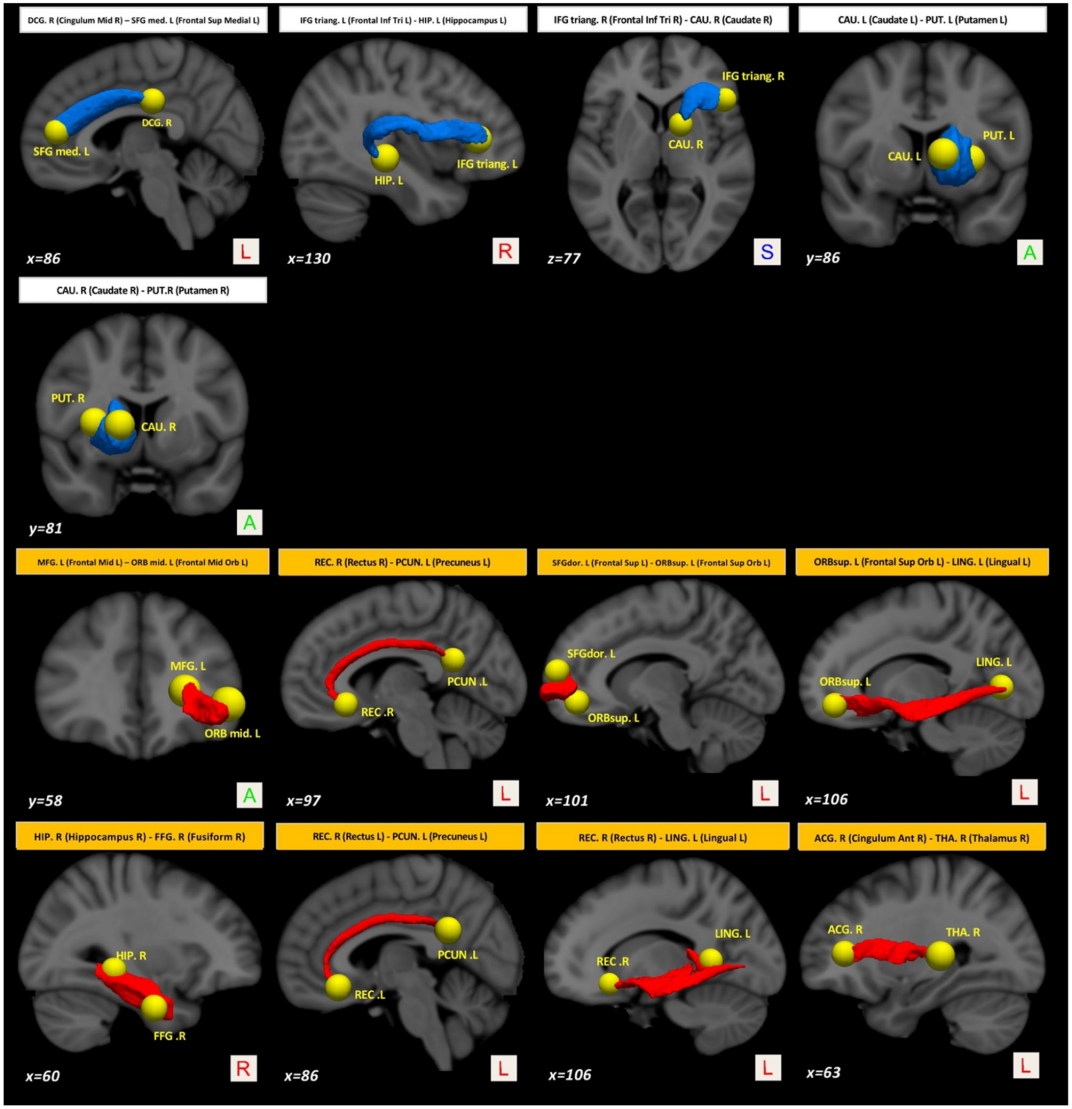
Tract reconstructions corresponding to structural connectivity differences in PD between baseline and after 36 months. The tracts were reconstructed by *connectome2tck* (MRtrix3) in the native space for all HC subjects, converted to track-weighted image by *tckmap* (MRtrix3), and normalized and averaged in the MNI 1mm standard space. For the visualization, Trackvis software (https://www.trackvis.org) was used. The blue tracts show higher connectivity in PD at the baseline. The red tracts show higher connectivity in PD after 36 months.

On the other hand, compared with the baseline, other paths showed a decrease in structural connectivity (Figure 4, paths in blue color) with a medium effect-size (between 0.568 and 0.685). The main WM areas connected to this trend are the anterior limb of internal capsule, anterior corona radiata, and the superior fronto-occipital fasciculus (see Table 3). Figure 5 shows the tract reconstructions for this analysis.

**Figure 6:**
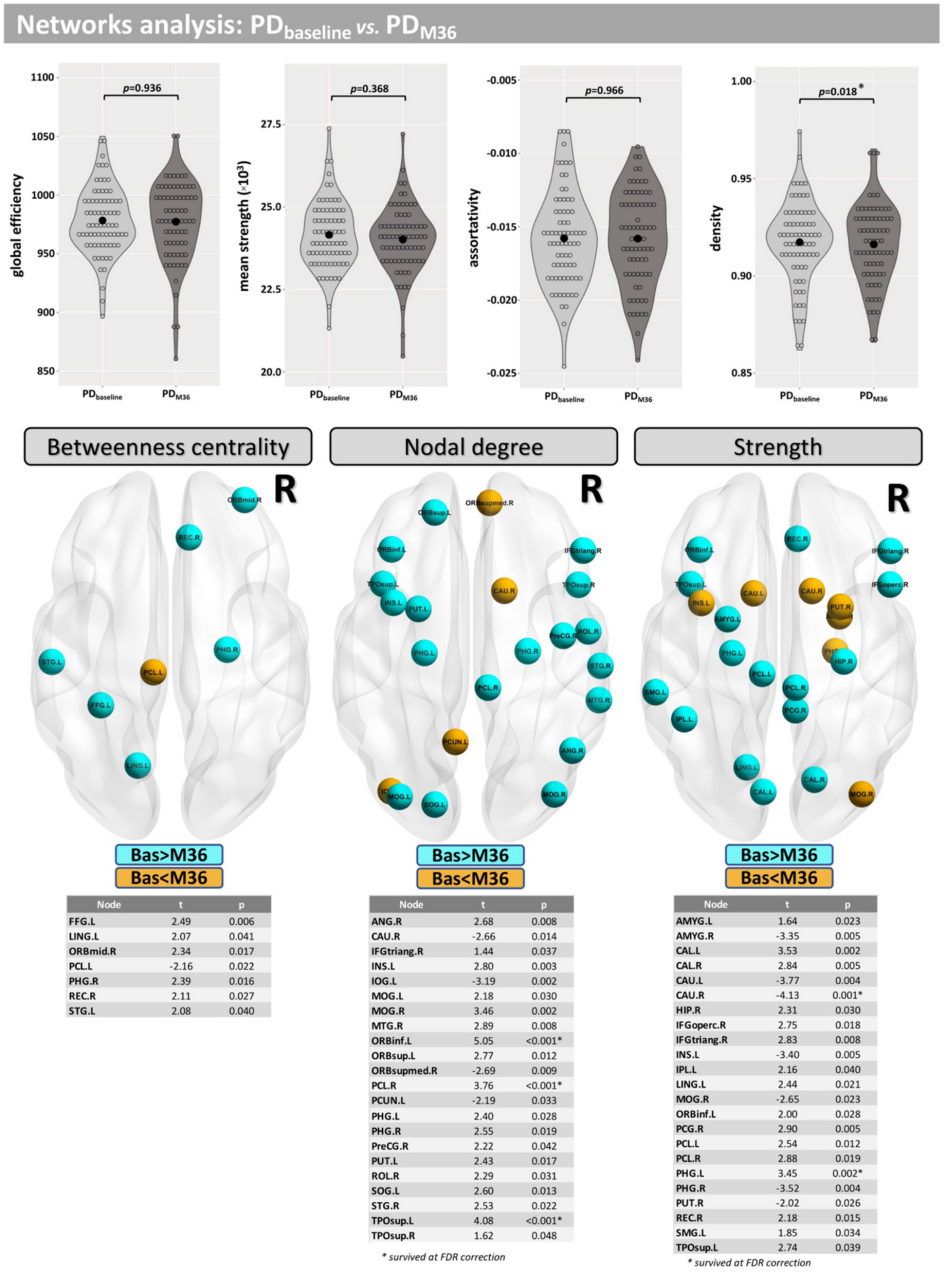
Network analysis for the longitudinal part of this study. Differences between baseline and M36 were found for the network density and in several nodes for “betweenness centrality,” “nodal degree,” and “strength.”

Compared with baseline, participants with PD displayed lower network density after 36 months, but this did not survive correction for multiple comparisons (*p* = 0.018-uncorrected). Additionally, differences were found at FDR <0.05 in betweenness centrality (mainly located inside frontal, occipital, and temporal lobes), nodal degree (mainly located inside the caudate, insula, putamen, occipital, and temporal lobes) and the strength (mainly located inside the caudate, insula, putamen, thalamus, occipital, and temporal lobes) (Figure 6).

### Correlations between structural connectivity and MDS-UPDRS/MoCA scores

**Figure 7:**
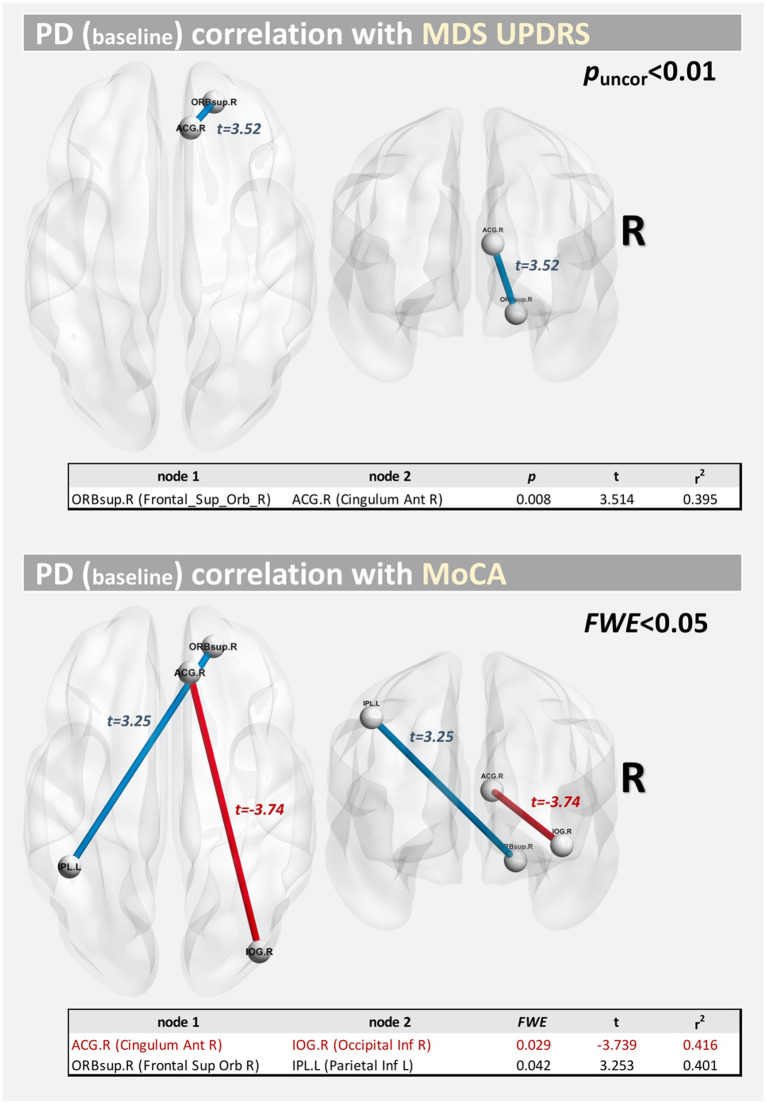
Correlations between structural connectivity and MDS-UPDRS/MoCA score at baseline.

Figure 7 shows the correlations at baseline between structural connectivity and MDS-UPDRS-III/MoCA scores. Significant correlations (at FWE <0.05) were found for MoCA, between the right anterior cingulate gyrus part of the cingulum (ACG.R) and the right inferior occipital gyrus (IOG.R) (*t* = −3.739; FWE = 0.029) and between the orbital part of the right superior frontal gyrus (ORBsup.R) and the left inferior parietal lobule (IPL.L) (*t* = 3.253; FWE = 0.042). For MDS-UPDRS-III, we only found a correlation at *p*-uncorrected <0.05 between nodes ORBsup.R and ACG.R (*t* = 3.514; *p* = 0.008).

## Discussion

In the current study, we analyzed structural connectivity and WM networks in participants with PD using both cross-sectional comparison with HCs and longitudinal evaluation across 36 months. We found significant differences in these metrics between PD and HCs, suggesting these metrics may reflect the impact of PD pathology on the brain's structure and networks. We also found longitudinal changes over a 36-month period, suggesting that structural and network metrics may be sensitive to disease progression. We discuss these results in more detail below.

Compared with the HC group, participants with PD showed lower structural connectivity in several brain areas, which were confirmed by a large effect-size. The PD group showed lower connectivity in several WM areas, such as, the corpus callosum (mainly in the genu), fornix, sagittal stratum, external capsule, cingulum, and uncinate fasciculus. Decreased structural connectivity in the corpus callosum might indicate degeneration of interhemispheric axonal connections between frontal areas. The corpus callosum is a critical structure for interhemispheric information transfer and thereby plays an important role in cognitive function (35). Indeed, other dMRI studies have shown that deterioration of the genu of the corpus callosum is linked to PD dementia (36), as well as executive and attention dysfunctions (37). Additionally, comparison of DTI metrics [such as FA and mean diffusivity (MD)] in the corpus callosum in PD has been shown to differentiate PD with and without cognitive impairment (38). In addition, structural alteration of the corpus callosum may be involved in predominant gait disorders (39) and impulse control disorders (40).

As already mentioned, other WM areas where connectivity was lower in PD participants were the fornix, cingulum, external capsule, and uncinate fasciculus. Altered DTI-related metrics have been found in these WM areas in PD using tract-based spatial statistics (TBSS) (41–45). Others have suggested that cognitive symptoms, such as episodic memory and visuospatial processing, as well as axial motor control, in individuals with PD may involve the cingulum (46). These findings might suggest that the lower connectivity inside the cingulum reported here might be responsible for the presence of such symptoms in this PD cohort (47). On the other hand, the fornix has important axonal connections with the subiculum of the hippocampus (48) and altered connectivity in this area is known to be associated with hippocampal damage (49, 50) as well as memory impairment in patients with PD (37). Together, these findings indicate that further analyses of structural connectivity metrics and their relationships with different PD symptoms are warranted.

In other WM areas, such as the right posterior limb of internal capsule and the left anterior corona radiata, the PD group exhibited higher connectivity than HC. Indeed, compared with the HCs, PD patients showed higher structural connectivity in only one path: between the left caudate and left medial frontal gyrus (orbital part). These findings are in line with the results reported by Mishra et al., which identified the presence of a distinctive PD-specific structural connectome, along with the unanticipated novel finding of increased structural connectivity between known PD-relevant brain regions including the caudate (15). These results support the hypothesis that structural network changes may underlie altered functional network capacity observed in PD. Additionally, the predominant structural connectivity differences occurred in the left hemisphere, suggesting lateral asymmetry. Several other studies have similarly shown that cortical atrophy (51) and nigral microstructural changes (52) are left lateralized in PD cohorts, with relative preservation of the right hemisphere until later in disease.

Altered connectivity between baseline and after 36 months was found in different paths at medium effect-size. It should be noted that these changes likely reflect a combined effect of both PD progression and aging, as changes in structural connectivity are known to occur with aging and may be attributed to brain reorganization that prioritizes selective connectivity to maintain function (53, 54). Interestingly, compared with PD at baseline, higher structural connectivity was found in PD subjects after 36 months inside 8 different paths (see Table 3 and Figure 4). WM areas with improved connectivity include the corpus callosum, fornix, and uncinate fasciculus. One explanation of these findings might be that increasing connectivity is indicative of compensatory changes, which have already been reported as compensation in later stages of the disease and the whole-brain/network scale that are probably due to the heterogeneous nature of PD (55, 56). However, on the other hand, we also found other 5 paths where PD patients at 36 months of follow-up had lower connectivity than at baseline.

Studies that have examined altered structural connectivity patterns in PD *via* graph theory analyses (57–59) have reported conflicting findings. This may be related to the differences in preprocessing and application of tractography methods (e.g., choice of tracking algorithm (probabilistic, deterministic, or other) and/or by the choice of edge weights) or could also be attributed to heterogeneity in PD populations. Here, we report modest between-group and within-group (PD) differences in network density (*p* < 0.05; uncorrected). The network density metric is equal to the numeric ratio of the actual connections to the possible connections. It indicates the degree of closeness of the relationships between the nodes in the network. In this study, the mean network density in the PD group was found to be lower than for controls. A similar trend was found for the longitudinal changes seen in PD.

Significant between-group and within-group differences were observed in betweenness centrality, nodal degree, and the strength in several nodes. Differences in the betweenness centrality metric may be associated with altered functional connectivity in PD (60) and were found in nodes mainly located in the frontal and temporal lobes. Differences in nodal degree were found mainly in caudate, frontal, occipital, and temporal lobes for HC vs. PD, as well as in the insula and putamen in the longitudinal comparison. Differences in strength were found mainly in nodes located in the caudate (baseline <Month 36), putamen, and amygdala, which are regions where similar changes have previously been observed in PD patients (61). Taken together, our results demonstrate that alterations in brain networks may be associated with PD and its progression.

Lastly, we also found significant correlations (FWE <0.05) at baseline between structural connectivity and MoCA score. These corresponded to two long-range connections, with an interhemispheric connection positively correlated with MoCA score and an intra-hemispheric connection negatively correlated with the MoCA score.

There are several strengths and limitations to this study. A major strength here is the use of a robust pre- and post-processing pipeline of the imaging data. One limitation is related to the single-shell data acquisition for dMRI, which has several drawbacks, including known inaccuracies related to partial volume effects (62) and difficulties to estimate the correct fiber orientation in voxels with complex fiber structure (e.g., crossing fibers) (63). In this study, we overcome these limitations by estimating the response function(s) for spherical deconvolution and the FOD (64) in order to more accurately estimate tractography. Additionally, the *tcksift2* (30) algorithm was applied to our tractographies to improve the accuracy of these reconstructions further. A final limitation is the lack of data available for longitudinal analysis of a HC group (not available in PPMI), and thus changes observed in the PD group cannot be attributed to the effects of PD pathology alone but are rather the combined result of aging and PD pathology. Using the data available, both group differences at baseline and longitudinal changes in the PD cohort were considered using advanced dMRI metrics, which is a strength of this study.

## Conclusion

In conclusion, we report altered structural and network metrics in several brain regions, such as corpus callosum, fornix, and cingulum, which showed lower connectivity in PD. We also report higher connectivity in the PD group, after 36 months, potentially due to compensatory processes as a result of the combined effects of PD pathology and aging. This data indicate that structural and network metrics could yield new diagnostic and progression markers for PD.

## Data availability statement

The data analyzed in this study was obtained from the Parkinson's Progression Markers Initiative (PPMI) database, the following licenses/restrictions apply: Investigators seeking access to PPMI data must sign the Data Use Agreement, submit an Online Application and comply with the study Publications Policy. Requests to access these datasets should be directed to PPMI, https://ida.loni.usc.edu/collaboration/access/appLicense.jsp.

## Ethics statement

Ethical review and approval was not required for the study on human participants in accordance with the local legislation and institutional requirements. The patients/participants provided their written informed consent to participate in this study.

## Author contributions

Conception, design, and original draft preparation: MB and AMS. Downloading data from PPMI database: MB. Data analysis and interpretation: MB, EK, and AMS. Manuscript writing, review, and editing: MB, EK, NJR, AM, MS, and AMS. All authors approved the final manuscript.
